# c-MET Regulates Myoblast Motility and Myocyte Fusion during Adult Skeletal Muscle Regeneration

**DOI:** 10.1371/journal.pone.0081757

**Published:** 2013-11-19

**Authors:** Micah T. Webster, Chen-Ming Fan

**Affiliations:** Department of Embryology, The Carnegie Institution for Science, Baltimore, Maryland, United States of America; Institut de Myologie, France

## Abstract

Adult muscle stem cells, satellite cells (SCs), endow skeletal muscle with tremendous regenerative capacity. Upon injury, SCs activate, proliferate, and migrate as myoblasts to the injury site where they become myocytes that fuse to form new muscle. How migration is regulated, though, remains largely unknown. Additionally, how migration and fusion, which both require dynamic rearrangement of the cytoskeleton, might be related is not well understood. c-MET, a receptor tyrosine kinase, is required for myogenic precursor cell migration into the limb for muscle development during embryogenesis. Using a genetic system to eliminate c-MET function specifically in adult mouse SCs, we found that c-MET was required for muscle regeneration in response to acute muscle injury. c-MET mutant myoblasts were defective in lamellipodia formation, had shorter ranges of migration, and migrated slower compared to control myoblasts. Surprisingly, c-MET was also required for efficient myocyte fusion, implicating c-MET in dual functions of regulating myoblast migration and myocyte fusion.

## Introduction

c-MET is a receptor tyrosine kinase activated by hepatocyte growth factor/scatter factor (HGF), its only known ligand, or in the absence of HGF by other factors such as Plexins [1]. The c-MET protein is post-translationally cleaved into two subunits; the extracellular alpha subunit is linked by disulphide bonds to the single pass transmembrane beta subunit. HGF activation of c-MET causes homodimerization and trans-phosphorylation at tyrosines (Tyr) 1234/1235 in the c-MET catalytic domain. Tyr 1234/1235 phosphorylation is critically important for subsequent phosphorylation of the intracellular multifunctional docking site that leads to recruitment of effectors and subsequent downstream signaling through multiple pathways [1]. HGF is typically a paracrine factor, expressed by mesenchyme to activate c-MET in the neighboring epithelia. The HGF/c-MET signaling axis enables invasive growth by regulating proliferation, survival, and migration. This axis is also crucial for skin and liver regeneration, and is commonly misregulated in many cancers [4-8]. 

During embryogenesis, myogenic precursor cells require c-MET for migration from the dermomyotome into the developing limb bud [2]. Limb mesenchyme derived HGF is required for de-epithelialization and migration of the muscle precursors [3]. Owing to multiple downstream signaling pathways, c-MET’s role in muscle development is multifaceted. A germline *c-Met* null mutation causes muscle precursors to remain in the dermomyotome but does not affect their proliferation [3], while a mutation that disrupts c-MET binding to one of its effectors, GRB2, inhibits muscle precursor proliferation only after cells migrate into the developing limb [9]. Thus, c-MET has different effects on muscle development depending on cellular context and effector binding.

*c-Met* is also expressed in adult quiescent satellite cells (SCs) [10], throughout myoblast activation and myocyte differentiation, and then down-regulated, but not absent in nascent myotubes [11]. In addition, HGF is involved during muscle regeneration [12], yet SCs are not the only *c-Met* expressing cell type present in the regenerating muscle milieu [13], making it unclear as to which cell types require c-MET signaling during the regenerative process. HGF has chemotactic [14] and mitogenic effects [15] on primary myoblasts, suggesting that c-MET activation could be important for muscle regeneration, in vivo. While these findings do imply that c-MET is involved in SC mediated muscle regeneration, c-MET’s role in muscle stem cell biology has not been addressed genetically.

 We hypothesized that SCs/myoblasts require c-MET function during muscle regeneration and devised a genetic strategy to test this hypothesis By conditional inactivation of c-MET specifically in SCs, we found that c-MET was required for SC mediated muscle regeneration in response to acute muscle injury. Curiously, c-MET was not required for SC activation, myoblast proliferation, or myocyte differentiation. We did identify a role for c-MET in enhancing SC/myoblast migration. We also uncovered an unexpected role for c-MET in regulating myocyte fusion during myotube formation, implicating c-MET in coordinating the migration of myoblasts and fusion of differentiated myocytes.

## Results

### SCs require c-MET during muscle regeneration

**Figure 1:**
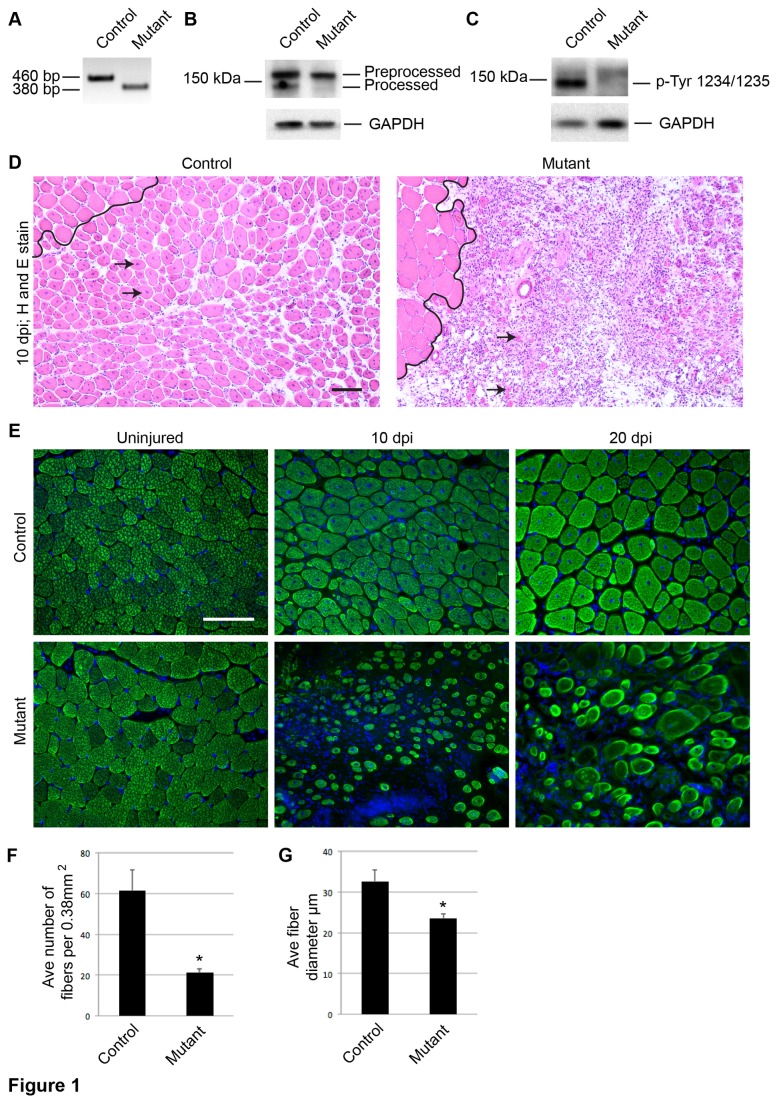
c-MET is required for SC mediated muscle regeneration. A-C) Control and mutant myoblasts were isolated by FACS via YFP fluorescence (cells were sorted only for data in A-C). A) RT-PCR analysis shows deletion of the 80bp exon 16 in mutant cells. B) Western analysis of total c-MET protein shows presence of both the preprocessed and processed forms of c-MET protein in control cells, while mutant cells lack the processed c-MET protein. C) Western analysis with a c-MET phospho Tyr 1234/1235 specific antibody shows c-MET activation is abolished in mutant cells. D) Hematoxylin and eosin staining of 10 dpi muscle sections shows robust muscle regeneration, indicated by cells with centrally localized nuclei, in control tissue, while mutant muscle shows fewer and smaller regenerated fibers (arrows, newly regenerated fibers; black line, boundary between injured and uninjured tissue; scale bar = 50 μm). E) Control and mutant muscle tissue showing IF against sarcomeric MHC in uninjured, 10, and 20 dpi muscle sections (scale bar = 50 μm). F) Quantification of regenerated fiber number per 0.38 mm^2^ area in 20 dpi control and mutant muscle sections (p = 3.36E-6 t test; N = 3 mice; 3 fields per mouse; error bars = SEM). G) Quantification of regenerated fiber diameter in 20 dpi control and mutant muscle sections (p = .0005 *t* test; N = 3 mice; 3 fields per mouse; error bars = SEM).

To eliminate c-MET function specifically in adult SCs, we combined a conditional *c-Met* allele containing loxP sites flanking exon 16 (*c-Met* ^*F*^ [5]), which codes for the ATP binding domain necessary for phosphorylation of Tyr 1234/1235, with the *Pax7-CreER* ^*T2*^ allele (*Pax7* ^*CE*^ [16]) for tamoxifen (TMX) inducible, Cre-mediated loxP recombination in SCs. Either a *LacZ* or *YFP* Cre-inducible lineage marker was included using the *Rosa26* locus (*R26R* ^*LacZ or YFP*^ [17,18]). TMX was administered to control (*Pax7* ^*CE/+*^ *; c-Met* ^*+/+*^ *; R26R* ^*LacZ or YFP*^; referred to as *R26R* ^*LacZ or YFP*^ control) and mutant (*Pax7* ^*CE/+*^ *; c-Met* ^*F/F*^ *; R26R* ^*LacZ or YFP*^; referred to as *R26R* ^*LacZ or YFP*^ mutant) adult mice for 5 consecutive days. A 10-day waiting period was implemented to allow for c-MET turnover. To determine efficacy of c-MET inactivation, we prepared bulk cultures from hind limbs of *R26R* ^*YFP*^ mutant and control mice. Myoblasts were isolated by FACS based on the YFP reporter. RT-PCR and Western analyses of these cells confirmed that inducible recombination effectively deleted *c-Met* exon 16 (Figure 1A), leading to the production of an unprocessed mutant protein (Figure 1B) with abolished phosphorylation at Tyr 1234/1235 (Figure 1C). Thus c-MET activity can be effectively eliminated in SCs using this genetic system.

We tested c-MET’s role in SCs during regeneration using a cardiotoxin (CTX) induced injury model. CTX was injected into the tibialis anterior (TA) muscle 10 days following TMX injections. At 10 days post injury (dpi), a benchmark for efficient muscle regeneration, regenerated fibers with centrally localized nuclei were numerous in control muscle. Mutant muscle showed a severe defect in muscle regeneration characterized by a drastic reduction of regenerated fibers and extensive fibrotic tissue (Figure 1D). Immunofluorescence (IF) for the muscle fiber marker, myosin heavy chain (MHC), showed that at 5 dpi (Figure S1A) and 10 dpi (Figure 1E), mutants had fewer and smaller (Figure S1B, C) regenerated muscle fibers compared to controls, directly demonstrating a requirement for c-MET in SCs for timely muscle regeneration in response to acute injury. At 20 dpi, mutant muscle maintained fewer and smaller regenerated fibers (Figure 1E-G), suggesting that c-MET was required for SCs to fully regenerate muscle following acute injury. 

**Figure 2:**
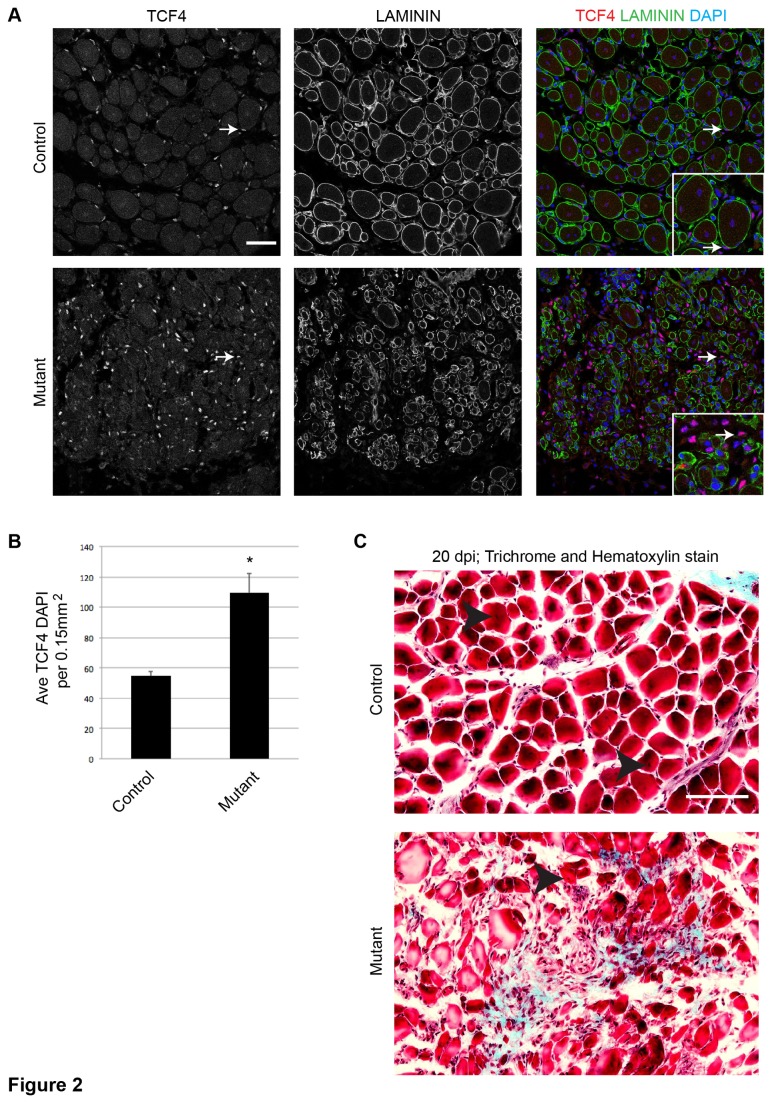
Increased density of TCF4+ fibroblasts and fibrosis during regeneration when SCs lack c-MET. A) Control and mutant muscle tissue showing IF against TCF4 and Laminin in 10 dpi muscle sections (arrows, TCF4+ DAPI+ nuclei external to Laminin enclosed fibers; inset shows enlarged regions; scale bar = 50 μm). B) Quantification of TCF4+ DAPI+ nuclei external to Laminin per 0.15 mm^2^ 10 dpi area (p = 1.19E-6 t test; N = 3 mice; 3 fields per mouse; error bars = SEM). C) Trichrome and hematoxylin staining of 20 dpi muscle sections shows fibrotic tissue (blue stain) throughout the injured area in mutant muscle (arrowheads, newly regenerated fibers; scale bar = 50 μm).

Extensive fibrosis is indicative of poor regeneration and is, in part, the product of TCF4+ muscle connective tissue fibroblasts. Normally, TCF4+ fibroblasts are present at low levels in uninjured muscle, become more numerous during the first few days of muscle regeneration, and decrease considerably by 10 dpi [19]. In the absence of a robust regenerative response to acute injury, TCF4+ fibroblasts remain numerous and lead to the accumulation of fibrotic tissue within the muscle [19]. Indeed, we observed an increase in the density of TCF4+ cells in 10 dpi, poorly regenerated mutant muscle sections compared to 10 dpi control muscle (Figure 2A, B). At 20 dpi, we observed increased levels of fibrosis in injured mutant muscle compared to control muscle (Figure 2C). These data further support that SCs require c-MET for a robust regenerative response to acute muscle injury, and that lack of c-MET in SCs correlates with increased fibrosis following injury.

### c-MET is not essential for SC survival, activation, proliferation, or differentiation

c-MET controls many cellular functions [1]. The defect in muscle regeneration (Figure 1D-G) could result from any number of SC dysfunctions. Therefore, we tested whether c-MET was needed for SC quiescence, activation, proliferation, and/or differentiation, as defects in any of these could impact regeneration. 

**Figure 3:**
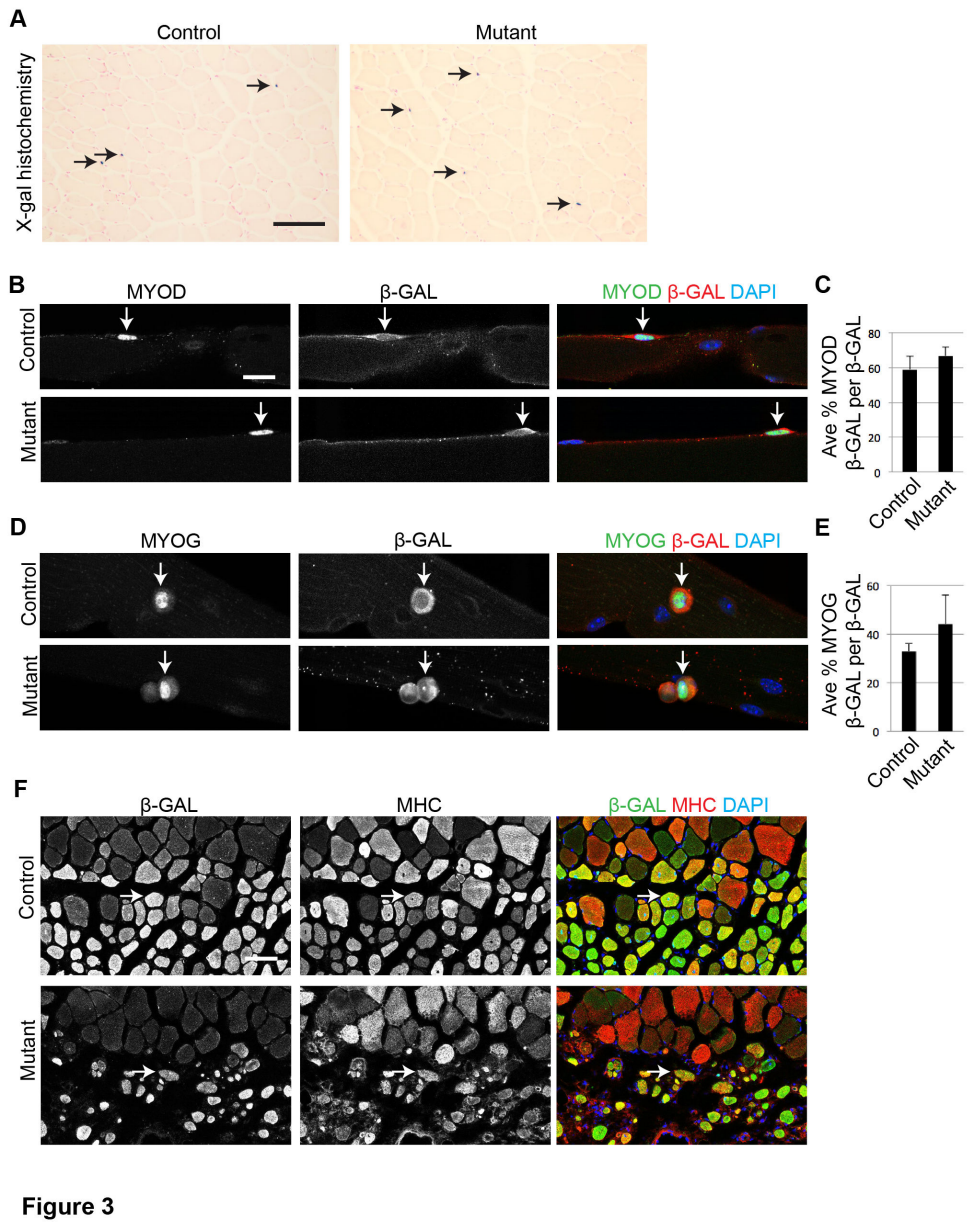
c-MET is not required for SC maintenance or differentiation. A) X-gal histochemistry applied to uninjured TA muscle from TMX injected control and mutant mice (arrows, X-gal positive cells; scale bar = 50 μm). B - E) Cultured single fibers from the EDL of control and mutant TMX injected mice. Fibers were cultured for B) 24 h and probed by IF for MYOD and β-GAL (arrows, MYOD+ β-GAL+ cell) or D) 72 h and probed by IF for MYOG and β-GAL (arrows, MYOG+ β-GAL+ cell; scale bar = 20 μm). Quantification of C) MYOD+ β-GAL+ per total β-GAL+ cells (p = .46 *t* test; N = 3 mice, n = 50 cells per mouse; error bars = SEM) and E) MYOG+ β-GAL+ out of total β-GAL+ cells (p = .80 *t* test; N = 3 mice; n = 100 cells per mouse; error bars = SEM). F) IF for β-GAL and MHC applied to 10 dpi control and mutant TA muscle sections (arrows, MHC+ β-GAL+ cells; scale bar = 50 μm).

Premature SC activation leads to depletion of the SC population [20]. c-MET’s effect on SC quiescence was tested by X-gal histochemistry of uninjured muscle sections from *R26R* ^*LacZ*^ control and mutant mice. Beta-galactosidase positive (β-GAL+) SCs were distributed in similar densities in controls and mutants (Figure 3A). In addition, the abundance of quiescent SCs on single fibers from extensor digitorum longus (EDL) muscle were not different among control and mutant fibers (2.4 ± .4 and 2.2 ± .3, respectively; p = .95 *t* test; N = 3 mice; n = 9 fibers per mouse). Thus, c-MET does not appear to be required during this timeframe to maintain quiescence of the SC population prior to activation. 

 SCs on cultured single fibers sequentially express markers of activation (MYOD) and differentiation (MYOGENIN (MYOG)) [21,22]. To test c-MET’s role during these stages, single fibers from control and mutant EDL muscle were harvested and cultured, and respective markers were assayed by IF. Similar rates of MYOD+ β-GAL+ (per total β-GAL+) cells were observed on control and mutant single fibers cultured for 24 h (Figure 3B, C). Likewise, similar rates of MYOG+ β-GAL+ (per total β-GAL+) cells were observed on control and mutant fibers cultured for 72 h (Figure 3D, E). Thus, c-MET signaling appears to not be required for SC activation or myoblast differentiation by this ex vivo assay. Consistently, β-GAL+ mutant cells expressed the terminal differentiation marker, MHC, in 10 dpi muscle tissue, demonstrating that mutant cells are able to activate and differentiate, in vivo (Figure 3F). Together, these data demonstrate that c-MET is not required for SC progression through hallmark stages of myoblast activation and myocyte differentiation, either ex vivo on single fibers or in vivo in response to CTX induced injury. 

**Figure 4:**
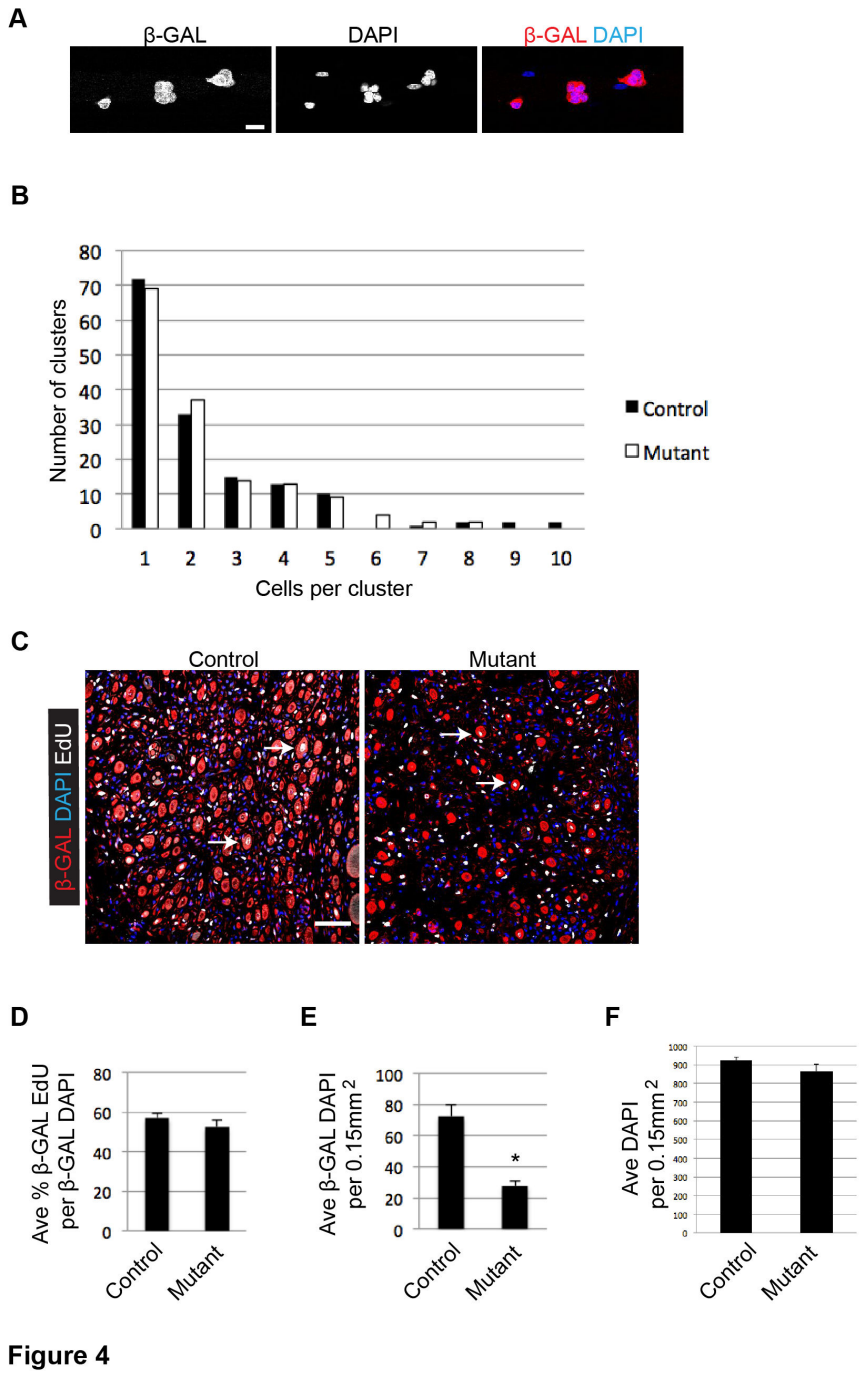
c-MET does not affect myoblast proliferation. A) Representative image of β-GAL+ cells in clonal clusters on EDL single fibers cultured for 72 h (scale bar = 20 μm). B) Histogram showing the number of β-GAL+ cells per cluster on control and mutant EDL single fibers cultured for 72 h (N = 3 mice; n = 50 cells per mouse). C) Representative images of 5 dpi TA muscle from control and mutant mice, probed by IF for β-GAL and EdU and stained with DAPI (arrows, β-GAL+ EdU+ cells; scale bar = 50 μm). D) Quantification of β-GAL+ EdU+/β-GAL+ DAPI+ cells in 5 dpi TA muscle sections (p = .36 *t* test; N = 3 mice; n= 866 for control, 333 for mutant; error bars = SEM). E) Quantification of β-GAL+ DAPI+ cells per 0.15mm^2^ injured TA muscle area (p = .02 *t* test; N = 3 mice; n = 4 injured muscle fields per mouse; error bars = SEM). F) Quantification of DAPI+ cells per 0.15mm^2^ injured TA muscle area (p = .18 *t* test; N = 3 mice; n = 4 injured muscle fields per mouse; error bars = SEM).

The mutant SC’s inability to robustly regenerate new muscle fibers (Figure 1D-F) could result from reduced SC proliferation. Two methods were used to assess SC proliferation. First, single fibers from *R26R* ^*LacZ*^ control and mutant mice were cultured for 72 h, during which time myoblasts proliferate and accumulate in clonal clusters (Figure 4A). Using cluster size as a metric for proliferation, we found very similar expansion profiles between control and mutant SCs (Figure 4B). Second, we assessed proliferation in vivo. *R26R* ^*LacZ*^ mutant and control mice were injected with TMX and injured as described. Mice were then injected once a day with 5-ethynyl-2′-deoxyuridine (EdU) from 2 to 5 days post injury, when the myoblast proliferative rate is highest following injury. Quantification of β-GAL+ EdU+ cells (including single cells and fibers) showed that, of the total β-GAL+ DAPI+ cells in the injured area, both control and mutant myoblasts had similar rates of EdU incorporation (β-GAL+ EdU+/β-GAL+ DAPI+; Figure 4C, D). While proliferation was comparable between mutant and control myoblasts, the number of β-GAL+ DAPI+ cells per injury field was greatly reduced in mutant muscle sections (Figure 4E). Total DAPI+ nuclei per injured area were similar between control and mutant (Figure 4F), suggesting that c-MET was required for SC accumulation at the injury site. Because *c-Met* is uniquely essential for muscle precursor migration during embryogenesis [2], we suspected that *c-Met* could contribute to myoblast migration during myoregeneration. 

### c-MET regulates myoblast migration

**Figure 5:**
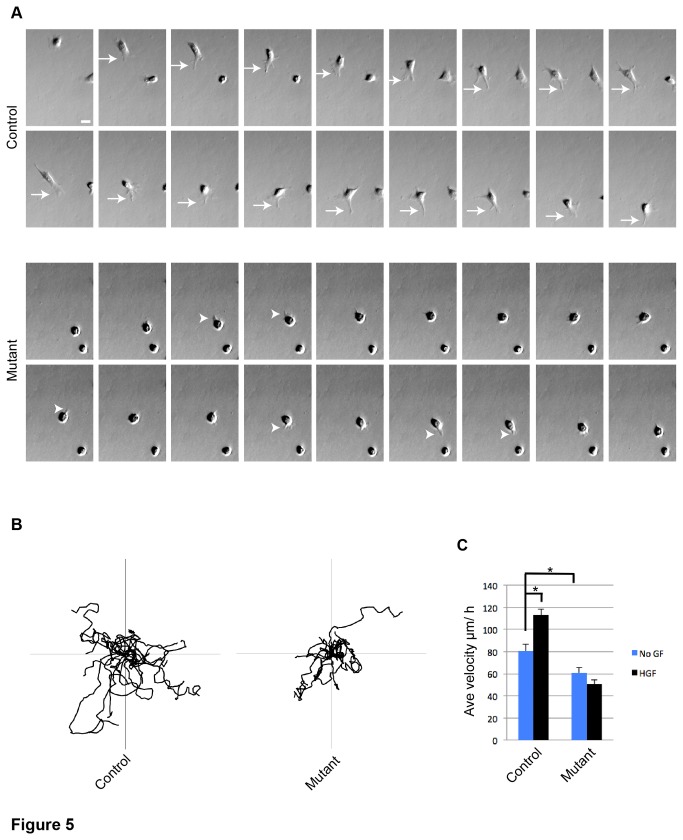
c-MET plays a role in cell morphology, distance traveled, and velocity during myoblast migration. A) Sequence of Hoffman modulation contrast (HMC) images showing live myoblasts in migration media. Images of the same cells were captured every 4 minutes for 68 minutes (arrows, lamellipodia in the control cell; arrowheads, membrane protrusions in the mutant cell; scale bar = 20 μm. B) Traces showing migratory paths of 15 myoblasts. Cell positions recorded every 4 minutes for 6 h (Axes = ± 300 μm). C) Average velocities of myoblasts in migration media without (no GF) or with supplement of HGF at 4 ng/ml. Images were taken once every 4 minutes, velocity measurements were recorded over 6 h. (p < .02 *t* test; N = 3 mice; n = 10 cells per mouse; error bars = SEM).

 An inability of mutant myoblasts to migrate from uninjured areas of the muscle into the injured area may explain the poor regenerative phenotype (Figure 1D-G). To assess c-MET’s role in myoblast migration, myoblast cultures were prepared from *R26R* ^*LacZ*^ control and mutant mice, and enriched in cultures by differential plating as assessed with a β-GAL fluorescent substrate, Imagene Green, and confirmed to contain ~90% marked myogenic cells for tracking (Figure S2). Cells were placed in migration media (see materials and methods), and cell migration was monitored by live cell imaging. We analyzed 3 aspects of cell migration: cell morphology, migratory distance from point of origin, and velocity. Individual control myoblasts showed sustained lamellipodia formation at the cell’s leading edge, determined by the direction of migration (example in Figure 5A top series; Video S1 for migrating control cells). By contrast, mutant cells had shorter, and less frequent lamellipodia formation (example in Figure 5A bottom series; Video S2 for migrating mutant cells). Traces of migratory paths from cells imaged for 6 h showed decreased migratory distances from point of origin in mutant cells compared to control cells (Figure 5B). Average velocity was calculated for individual myoblasts cultured for 6 h. In the absence of exogenous HGF, control cells migrated faster than mutant cells, suggesting that autocrine HGF signaling [23], or activation of c-MET by another factor [24], enabled optimal myoblast migration (Figure 5C blue bars). Exogenous HGF increased migratory velocity in control cells. Mutant cells, however, did not migrate faster when exposed to HGF, consistent with c-MET inactivation in the mutant (Figure 5C black bars) and c-MET being the exclusive receptor to mediate HGF’s chemotactic effect.

**Figure 6:**
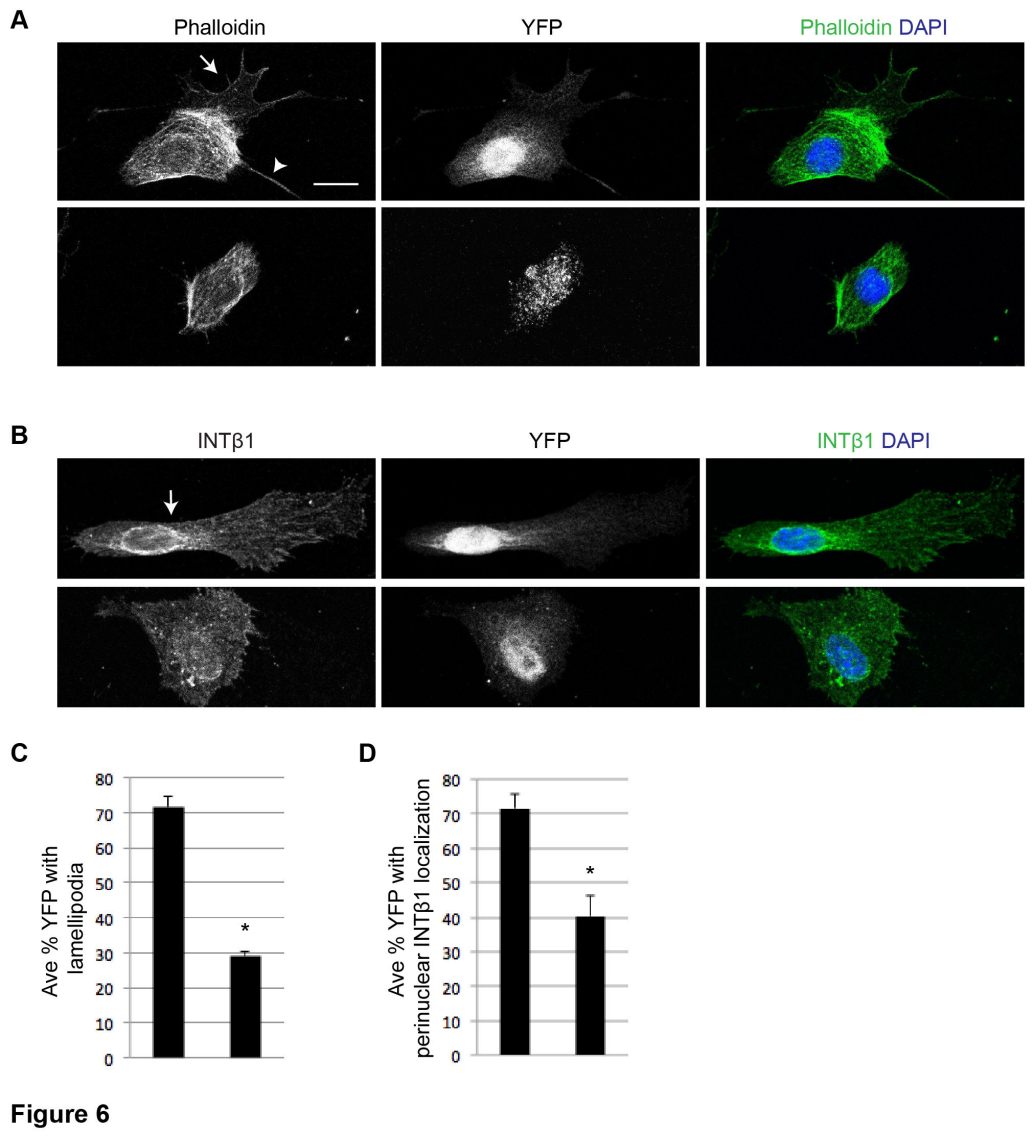
c-MET contributes to lamellipodia formation and peri-nuclear INTβ1 localization. A) Representative images of cells stained with phalloidin and DAPI with IF for YFP (arrow, lamellipodia; arrowhead, long actin protrusion; scale bar = 5 μm). B) Representative images of cells with IF for INTβ1 and YFP (arrow, peri-nuclear INTβ1 localization; scale same as in (A)). C) Average percentage of YFP+ cells with phalloidin stained lamellipodia as example shown in (A)(p = .00029 *t* test; N = 3; n = 120 cells; error bars = SEM). D) Average percentage of YFP+ cells with peri-nuclear INTβ1 as example shown in (B) (p = .013 *t* test; N = 3; n = 120 cells; error bars = SEM).

 The effect of c-MET on myoblast migration is reminiscent of c-MET’s known role in regulating the cytoskeleton [8]. Indeed we found that mutant myoblasts frequently lacked phalloidin stained lamellipodia (Figure 6A, C). We also probed for INTEGRIN-β1 (INTβ1) in migrating myoblasts; integrins are present in lamellipodia and important for cell migration [25], are known to interact with c-MET [1], and INTβ1 affects migration in myoblasts [26]. IF showed INTβ1 distribution throughout lamellipodia and peri-nuclear regions in control myoblasts, while mutant cells showed a loss of organized distribution resembling lamellipodia and peri-nuclear staining, which indicated aberrant INTβ1 distribution in mutant myoblasts (Figure 6B, D). 

Together, these data support that adult myoblasts require c-MET function for efficient, but not absolute, motility. Myoblasts seem to utilize c-MET signaling for enhanced migration, regardless of an exogenous HGF source, and HGF stimulated myoblast motility requires c-MET. The defect in mutant myoblast migration is likely due, in part, to actin cytoskeletal dynamics and INTβ1 distribution. 

### c-MET mutant myoblasts are defective in myoblast fusion

**Figure 7:**
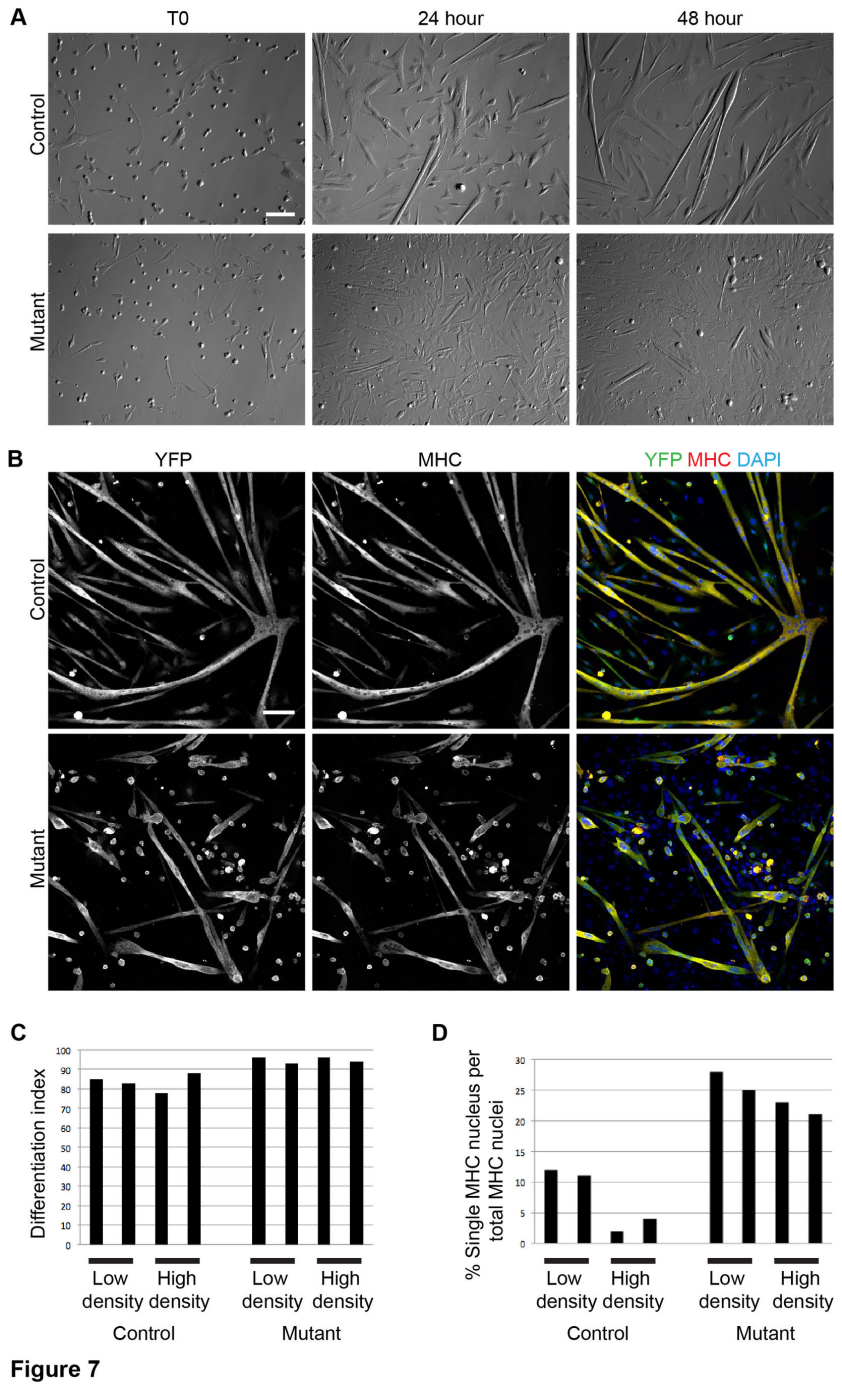
Myoblasts require c-MET for efficient fusion. A) HMC images showing the same fields of low-density, live, myoblasts in differentiation media at T0, 24, and 48 h. (scale bar = 100 μm). B) Representative images of high-density *R26R* ^*YFP*^ control and mutant myoblasts after 3 days in differentiation media. Cells were probed by IF for MHC and YFP and stained with DAPI (scale bar = 100 μm). C) Differentiation index (MHC+ YFP+ nuclei per YFP+ nuclei) and D) Index of unfused cells (MHC+ YFP+ cells with single nucleus per total MHC+ YFP+ nuclei) was quantified in 3-day differentiation cultures. Two independent cell cultures were tested for each condition. Low-density cells were plated at 13,000 cells per cm^2^; high-density cells were plated at 40,000 cells per cm^2^. At least 110 MHC+ YFP+ cells were counted for each culture and condition.

 In addition to migration, the cytoskeleton is important for myocyte fusion [27-29]. Myocytes will readily fuse and form myotubes when grown in differentiation media (see materials and methods). To investigate a possible role for c-MET during myotube formation, mutant and control myoblasts were grown in differentiation media and imaged at 24 and 48 h time-points. We observed a transient flattening morphology of mutant cells at 24 h, and fewer, smaller, and dysmorphic myotubes formed in the mutant culture compared to the control at 24 and 48 h (Figure 7A). These data suggest a requirement for c-MET during myotube formation.

To determine if the mutant myocytes had a fusion or a terminal differentiation defect, *R26R* ^*YFP*^ control and mutant myoblasts were grown in differentiation media for 3 days and then probed by IF for MHC and YFP. Control myocytes fused to form multinucleated MHC+ myotubes after 3 days (Figure 7B). Mutant myocytes showed a qualitative fusion defect characterized by many rounded MHC+ YFP+ cells containing a single nucleus, as well as dysmorphic MHC+ myotubes (Figure 7B). To determine whether the rounded cells were apoptotic, we assayed for cleaved CASPASE-3 (CAS3) and compared 3-day mutant and control differentiating cultures (Figure S3A, B). Although there were slightly more CAS3+ YFP+ cells in mutant cultures, these were unlikely to account for the high percentage of unfused MHC+ cells present in the mutant cultures (Figure 7D). In addition, we did not detect CAS3+ MHC+ double positive cells (Figure S3C), consistent with previous findings that terminally differentiated muscle cells do not undergo apoptosis [30]. We next suspected that the mutant migration defect could affect myocyte fusion simply because cells would not be able to migrate long distances towards one another as effectively as controls. Therefore we tested both high and low-densities of cells in the fusion assay. Differentiation indices showed a slight increase in MHC+ mutant cells per total YFP+ cells compared to control cultures under both densities (Figure 7C), indicating that differentiation was not compromised in mutant cells under these conditions. However mutant cultures had > 2 fold more unfused MHC+ cells compared to controls at both low and high cell densities (Figure 7D). It is unlikely that the minimal number of other cell types in differentiating cultures physically blocked fusion of mutant myocytes as many MHC+ YFP+ cells with a single nucleus were found in close proximity to each other (Figure 7B). These data suggest that long-range migration did not affect mutant fusion rates and that c-MET was required for optimal myocyte fusion and myotube formation, either directly or indirectly. 

## Discussion

We have demonstrated a genetic requirement for *c-Met* in SCs during muscle regeneration in response to acute injury in vivo. Previous studies using neutralizing antibodies to c-MET ‘s ligand, HGF, have shown that blocking this signal axis hinders adult muscle regeneration in vivo [12]. Because there are other muscle resident cell type(s) expressing c-MET [13], it has not been clear as to which cells required c-MET activation. Our SC specific inactivation of *c-Met* function provides direct evidence that SCs are at least one cell population requiring c-MET activity during muscle regeneration. Employing various in vivo and in vitro assays coupled with lineage marker analyses, we were able to determine that *c-Met* mutant SCs were not defective in proliferation and differentiation. Rather, they were defective in basal, as well as HGF-stimulated, motility in vitro, accompanied by reduced lamellipodia formation and abnormal intracellular distribution of INTβ1. We also document an unexpected defect in mutant myoblast fusion in vitro, suggesting a role for c-MET in this process. These primary defects (migration and fusion) in mutant SCs help explain the mutant’s muscle regeneration defects in vivo, i.e. reduction of myogenic cell numbers as well as reduced sizes and numbers of regenerated myofibers. 

### c-MET-HFG signaling axis in SC activation and proliferation

By assaying rates of EdU incorporation among lineage marked myogenic cells in vivo, we found proliferation to be similar between c-*Met* mutant and control cells in the injured/regenerative area, despite there were considerably fewer myogenic cells in the mutant (Figure 4). We also did not detect a significant difference in clonal expansion of SCs on single fibers between mutant and control cells, an assay presumably not dependent on cell migration (Figure 4). Our data are surprising as administration of HGF is well known to accelerate SC activation and to enhance SC proliferation [11,13,31-34]. One possible explanation is that other activation and proliferation factors, in vivo and in culture media additives, mask the requirement for HGF-c-MET signaling. FGF family members are prime candidates for such a compensatory role, as they also utilize receptor tyrosine kinases like c-MET. In addition, bFGF is a potent proliferation factor for SCs in culture [35,36] and FGF signaling is required for SC activation or proliferation during muscle regeneration [37]. Of note, a *c-Met* null mutant was reported not to affect the proliferation, only the delamination and migration of myogenic progenitors in the dermomyotome [3]. Additionally, the same *c-Met* conditional allele used in our study was first used in liver regeneration assays. While mutant hepatocytes did not respond to HGF in vitro, they were not defective in proliferation after injury in vivo [5]. Our data provide yet another cell context in which c-MET is not required for proliferation. Given that the recombined conditional *c-Met* allele produces an unprocessed mutant protein incapable of Tyr 1234/1245 phosphorylation (Figure 1), a presumed signaling null, our data support that c-MET signaling in SCs does not play an essential role in their activation and proliferation, and should reshape previous notions for this signaling axis in SC biology.

### c-MET-HFG signaling axis in SC migration

Findings from our live tracking of lineage marked myoblasts do support a role for c-MET in enabling efficient myoblast migration. We also found, intriguingly, that c-MET function is required for optimal “basal” migration (i.e. *c-Met* mutant cells do migrate) in the absence of exogenous HGF (Figure 5). One possibility is that HGF autocrine signaling enables myoblast migration [23]. This may seem contradictory to studies that conclude HGF is a chemoattractant for myoblasts [14,38] as there would be no gradient for chemoattraction. However, since exogenous HGF can induce migration (this study and [14,38]), c-MET signaling is not saturated by a cell autonomous source of HGF, leaving open the possibility for myoblasts to detect an exogenous HGF gradient. Another possibility for the reduced basal migration of mutant SCs is that c-MET is involved in modulating another cellular component(s) for motility in a HGF-independent manner [24]. 

HGF signaling leads to decreased actin stress fibers and cell motility [39] by activating the small GTP-binding proteins Ras and Rac [40]. In myoblasts, HGF induced motility requires the actin remodeling proteins WAVE-2 and N-WASP [41]. Consistent with c-MET’s role in actin reorganization during migration, we observed *c-Met* mutant cells forming smaller cellular extensions during migration, had shorter ranges of migration from point of origin, and migrated slower than controls (Figure 5). In addition, *c-Met* mutant myoblasts commonly lacked elaborate lamellipodia, consistent with decreased motility. Mutant cells also had a lower frequency of peri-nuclear INTβ1 localization, compared to control cells (Figure 6.). It was recently documented that INTβ1-mediated migration involved the recycling of INTβ1 from the plasma membrane to a peri-nuclear compartment [42]. We thus propose a mechanistic requirement for c-MET in INTβ1 recycling for efficient migration. 

Our in vitro findings help explain the muscle regeneration defect observed in the SC-specific *c-Met* mutant in vivo: Mutant myoblasts had a reduced capacity to migrate from uninjured regions of the muscle, thus leading to reduced presence of myogenic cells in the injured area. In this regard, there must be release of HGF at or near the injury site to stimulate SC migration via c-MET signaling. Thus, c-MET’s role in the SCs for adult muscle regeneration is similar to its role in dermomyotomal cell migration [2,3]. Another associated in vivo defect in the SC-specific *c-Met* mutant is the increase of TCF4+ fibroblasts at the injured area (Figure 2). SCs and fibroblasts are known to expand in close proximity during early regeneration, and efficient muscle regeneration eventually leads to the recession of fibrogenic cells. Although the molecular mechanism(s) controlling their interplay is not well understood, ablating the majority of SCs results in prolonged increase of TCF4+ fibroblasts at the poorly regenerated injury site [19]. We suggest that reduced supply of SCs, either by their ablation or by their compromised migratory capability, elicit prolonged accumulation of fibroblasts, which eventually participate in the formation of fibrosis. 

### c-MET-HGF signaling axis in myogenic differentiation and fusion

We documented the ability of *c-Met* mutant SCs to differentiate in vitro (Figure 7) and form MHC+ regenerated fibers in vivo (Figure 3), similar to control SCs. On the other hand, administration of HGF has been shown to delay SC differentiation in vitro and can inhibit muscle regeneration in vivo [11,43]. These “ligand gain of function” observations are not inconsistent with our “receptor loss of function” data. It seems likely that stimulation of SC proliferation and inhibition of SC differentiation by HGF are linked processes. Since we did not find an essential role for c-MET in SC proliferation, it is not surprising we also did not detect its role in impacting differentiation. It is nevertheless possible that the in vivo environment and the in vitro culture media mask such a requirement for c-MET in our loss of function experimental paradigm. 

We also found, unexpectedly, that myoblasts fused less frequently in the absence of c-MET function (Figure 7). Contrasting to development, myogenic precursors that lack c-MET never delaminate from the dermomyotome, which precluded subsequent assay for muscle cell fusion in the limb [2]. In vitro, migrating myoblasts are known to slow down prior to initiating fusion and forming myotubes [44,45]. Furthermore, myotube formation has been described as occurring in two steps. First myoblasts will fuse to establish nascent myotubes, which then recruit more myoblasts for a second round of fusion [29]. We note that *c-Met* mutant myoblasts initiated fewer primary fibers than control myoblasts after 24 h in differentiation media (Figure 7). Even at 48 h, mutant fibers remained small and short (Figure 7), strongly suggesting that the first step of fusion is defective. This defect in the first step of fusion makes it difficult to determine whether it is also directly involved in the second step of fusion. Intriguingly, the temporal expression profile of differentiating myoblasts showed a decline in c-*Met* levels after 48 h, and a drastic increase of *Hgf* levels between 48-96 h [11], highly suggestive of a role for HGF produced by nascent myotubes to recruit lower *c-Met* expressing myoblasts for the second round of fusion. It therefore remains possible that *c-Met* is directly involved in regulating both steps of fusion. On the other hand, we cannot rule out that the fusion defect of *c-Met* mutant myoblasts documented here is a secondary consequence of their migration defect as fusion events rely on cells coming in contact via migration. Results from our high-density cell fusion assay would suggest that the defect of *c-Met* mutant SCs is independent of their long-range migration defect (Figure 7). Because *c-Met* mutant SCs are mobile but defective in the proper distribution of membrane and cytoskeletal components, they may not be able to align properly for fusion. As mentioned above, *c-Met* mutant cells exhibit a defect in INTβ1 distribution (Figure 6). Because INTβ1 is required for fusion [46], its abnormal distribution may contribute to the fusion defect observed in *c-Met* mutant cells. 

In this study, we utilized a genetic approach to clarify the role of c-MET in muscle regeneration by conditional inactivation of *c-Met* specifically in the SC. We conclude that c-MET activity in the SCs is important for their migration and fusion, but not for their activation, proliferation or differentiation. Equally important is that our data provide critical information for re-evaluating various presumed roles of c-MET/HGF in previous studies. 

## Materials and Methods

### Animals and tissue harvest

The *c-Met* ^*F/F*^ mouse (FVB;129P2-*Mettm1Sst*/J) was a gift from Dr. Snorri S. Thorgeirsson at the National Cancer Institute, National Institutes of Health, Bethesda (described in [5]). The *Pax7* ^*CE*^ allele (B6;129-*Pax7* ^*tm2.1*(*cre/ERT2*^)*Fan*/J) has been described [16]. Both *R26R* ^*lacz*^ (B6.129S4-*Gt*(*ROSA*)*26Sortm1Sor*/J) [17] and *R26R* ^*YFP*^ (B6.129X1-*Gt*(*ROSA*)*26Sor* ^*tm1*(*EYFP*^)*Cos*/J) [18] reporter mice were obtained from the Jackson Laboratory. Appropriate mating schemes were used to generate mice of mixed background with genotypes described in the text. Littermates were used as controls whenever possible. Mice were genotyped by PCR (primers described in [5,16]. Adult female and male mice, 2-6 months of age, were used (with the number of males or females matching between control and mutant for experiments described, with the exception that only males were used for single fiber analyses). We performed experiments on each set (same number of controls and mutants) of animals in parallel. Due to the time needed to accumulate animals of desired genotypes from genetic crosses for side-by-side experimentation, animals between 2-6 months were used. Mice were given Tamoxifen (TMX)(Sigma; diluted to 20 mg/ml in corn oil (Sigma)), 3 mg per 40 g body weight per intraperitoneal injection, once a day consecutively for 5 days. All experiments were conducted 10 days after the final injection. Mice were anesthetized using 2,2,2-Tribromoethanol (Sigma), which was dissolved in 2-methyl-2-butanol (Sigma) as 100% (w/v) stock solution. Working dilution was made fresh monthly in PBS at 2.5 %, filtered by 0.22 μm syringe filter (VWR), stored at 4°C, and used at 10 μl per gram of body weight by intraperitoneal injection. For injury, 50 μl of 10 μM cardiotoxin (CTX)(Sigma) was injected with an insulin syringe (BD) into TA muscles. EdU (Invitrogen) was given by intraperitoneal injection at 0.1 mg per 20 g bodyweight per injection, once a day from 2 to 5 days after injury. Mice were sacrificed by cervical dislocation and TA muscles were harvested by carefully cutting the tendons to remove the whole muscle intact, fixed for 8 minutes in ice cold 4% paraformaldehyde (EMS) in phosphate buffered saline (PBS; Gibco), incubated overnight in each 10%, and 20% sucrose PBS, then mounted on cork using Tragacanth (Sigma) and flash frozen for 30 seconds in 2-Methylbutane (Sigma) cooled by liquid nitrogen. This study was carried out in strict accordance with the recommendations in the Guide for the Care and Use of Laboratory Animals of the National Institutes of Health. All mouse manipulations were approved by the Institutional Animal Care and Use Committee (IACUC) of the Carnegie Institution for Science (Permit number A3861-01).

### Cell culture, FACS, and RT-PCR

Myoblasts were prepared by bulk culture from total hind limb muscles. Muscles were dissected, minced, and incubated in 0.2% Collagenase Type I (Sigma) in DMEM at 37°C with gentle shaking for 1.5 h. Muscle was then triturated in 10% FBS in DMEM, washed with PBS, and incubated in 0.2% dispase (Gibco) in DMEM at 37°C with gentle shaking for 30 minutes. Cells were filtered through a 70 μm cell strainer, plated on Matrigel (BD Biosciences) coated 10 cm tissue culture plates in 20% FBS, 5% horse serum, 1% Penn/Strep (Invitrogen), 1% Glutamax (Invtrogen), DMEM (referred to as proliferation media, as opposed to differentiation and migration medias (see below)) and incubated (37°C, 5% CO_2_). Cells were incubated for 3 days, removed from plates by .25% Trypsin/EDTA (Life Technologies) treatment for 7 min, differentially plated in proliferation media on uncoated dishes for 1 h to remove non-myogenic cells (cultures maintained with a cell population that was ~90% marked myogenic by Imagene Green analysis, described below), and then returned to Matrigel coated plates. Cells were maintained in proliferation media for up to 3 passages with differential plating between passages. Myotube formation was infrequent in these cultures. Cells were sometimes frozen within 5 days of harvesting and then thawed once for future use. 

For FACS analysis, primary cells were cultured for 3 days, passaged once, and cultured for 2 more days to expand the myoblast population prior to sorting. FACS isolation based on YFP fluorescence was done with a BD FACS ARIA III and FACS Diva software, using the 488 channel and gating first for cell size using forward and side scatter, and then gating for YFP+ cells. We noted that our dissociation and culturing technique yielded similar quantities of SCs from FACS and differentially plated preparations. FACS purified cells were used immediately after sorting for RNA and protein isolation. RNA was isolated using the Arcturus PicoPure RNA isolation kit (Applied Biosystems), primers for *c-Met* RT-PCR were ttaggcaatgaggtgtcccac and cagccgtcagacaattggcac. 

Single fibers were prepared by carefully removing the Extensor Digitorum Longus (EDL) muscle making sure not to damage the tendon at either end of the muscle. EDLs were placed in 0.2% Collagenase Type II (Gibco), DMEM in a 37°C water bath with gentle shaking for 1 h. Media was replaced with pre-warmed DMEM, and single fibers were dissociated by gentle trituration (during which some SCs may have been liberated from the fibers) using flame-polished glass Pasteur pipettes that had been pre-coated with horse serum to limit fibers sticking to the glass. Fibers were cultured by suspension in proliferation media (incubated at 37°C, 5% CO_2_) in plates that had been coated with horse serum to limit fibers from sticking to the plastic. The presence of non-myogenic cells on cultured fibers was minimal (< 1%) as determined by immunofluorescence (IF) for the β-GAL lineage marker (see below for IF).

### Western blot

YFP marked myoblasts were prepared and isolated by FACS (described above), and protein was extracted from cell pellets using T-Per Tissue Protein Extraction Reagent (Thermo), 1mM PMSF, 1x Halt Phosphatase Inhibitor Cocktail (Thermo), Complete Protease Inhibitor Tablet (Roche). Pellets were disrupted using a Pestle (Kimble Chase), spun at 4°C in a microcentrifuge at maximum speed, and supernatant was added to 4x SDS sample buffer to achieve a final 1x concentration. Samples were boiled for 5 minutes and then separated by 7.5% SDS-PAGE (Bio-Rad) with Kaleidascope molecular mass marker (Bio-Rad). Western transfer to Immuno-blot PVDF Membrane (Bio-Rad) was done overnight at 4°C using a Bio-Rad mini-Protein II Transfer system. Membrane was blocked in 5% low-fat Carnation milk powder, 0.1% Tween 20 (Sigma), TBS for 1 h at room temperature, incubated with primary antibody in blocking solution overnight at 4°C (Goat anti-c-MET (R & D, AF527) 1:500, Rabbit anti-phospho-c-MET Y1234/1235 (Cell Signaling, 3077S) 1:500, Mouse anti-GAPDH (Chemicon, MAB374) 1:1000), washed in 0.1% Tween 20 TBS, 3x, 10 minutes each, incubated with secondary antibody for 1 h at room temperature (HRP conjugated anti-Goat (Invitrogen), anti-Rabbit (Invitrogen), and anti-Mouse (Millipore) at 1:10,000), washed in 0.1% Tween 20 TBS, 2x, 5 minutes each, followed by a third, 2 h wash, then ECL reaction using Femto Chemiluminescent Substrate (Thermo) and exposed to X-ray film. 

### Immunostaining and Quantification

10 μm sections of TA muscle, on SuperFrost Plus slides (Fisher) were permeabilized with 0.1% Triton-X 100 (Sigma), PBS for 20 minutes at room temperature, washed once with 0.05% Triton-X 100, PBS (PBS/T), and then incubated in Mouse IgG1 blocking solution from the M.O.M. Kit (Vector Lab), diluted in PBS/T at 1 drop per 1.5 mls, for 6 h or overnight at 4°C. Sections were then washed with PBS and incubated in 10% Normal Goat Serum (Genetex), 1% Blocking powder (Perkin Elmer), PBS/T (NGB) for 30 minutes at room temperature. Primary antibodies (Mouse IgG2_b_ anti-Sarcomeric Myosin (DSHB, supernatant, MF20) 1:20, Mouse IgG anti-Embryonic Myosin (DSHB, supernantant, F1.652) 1:20, Rabbit anti-β-GAL (Cappel, #55976, currently from MP Biomedicals) 1:5000, Mouse IgG2_a_ anti-TCF4 (Millipore) 1:250 (slides treated with antigen unmasking solution (Vector) by boiling for 10 minutes prior to TCF4 staining [19]) were diluted in NGB and applied to sections for 6 h or overnight at 4°C. Sections were washed with PBS/T, 3x, and then incubated in secondary antibodies, diluted in NGB, for 1 h at room temperature (Alexa Fluor 488 conjugated Anti-Mouse IgG1 1:1000, Alexa Fluor 568 conjugated Anti-Mouse IgG2_b_ 1:500, Alexa Fluor 488 conjugated Anti-Rabbit 1:1000, all from goat (Invitrogen), and anti-Chicken IgG Antibody DyLight 549 1:500 from goat (Rockland)). Slides were washed with PBS/T, EdU was detected using the Click-iT reaction kit (Invitrogen), incubated in DAPI 1μg per ml of PBS/T for 5 minutes at room temp, washed with PBS/T and then mounted with Fluoromount-G (SouthernBiotech). Muscle fiber diameter was measured as the shortest diameter, only cells with centrally localized nuclei were quantified. Imaged fields measured .38mm^2^.

Single fibers were fixed in 4% PFA for 8 minutes, washed in PBS, 3x, 5 minutes per wash, permeabilized with 0.5% Triton-X 100, PBS for 15 minutes, and then washed with PBS/T. Fibers were incubated in NGB overnight at 4°C, and then in primary antibody diluted in NGB overnight at 4°C (Mouse IgG1 anti-Pax7 [16] 1:20, Mouse IgG1 anti-MYOD (DAKO, M351201) 1:1000, Mouse IgG1 anti-MYOGENIN (DSHB, supernatant, F5D) 1:50, Rabbit anti-β-GAL 1:5000). Fibers were washed with PBS/T, incubated with fluorescent secondary antibodies, as described above, in NGB followed by DAPI, washed in PBS/T and mounted with Fluoromount G on SuperFrost Plus slides.

Myoblasts, cultured in 8-well chamber slides (Thermo), were fixed in 4% PFA, PBS for 8 minutes at room temperature, washed with PBS, permeabilized with 0.5% Triton-X, PBS for 15 minutes, washed with PBS/T, and incubated with NGB for 1 h at room temp. Primary antibody was applied in NGB for 2 h at room temperature (Mouse IgG2_b_ anti-Sarcomeric MYOSIN (DHSB, MF20) 1:20, Rabbit anti-GFP (Invitrogen, G10362) 1:500, Chicken anti-GFP (Aves) 1:500, Rat anti-INTEGRIN β1 (Millipore) 1:200, Rabbit anti-Cleaved CASPASE-3 (Cell Signaling) 1:200), followed by PBS/T, 3x, 5 minutes per wash. Alexa Fluor 488 phalloidin (Invitrogen) diluted 1:40 was applied to cells with fluorescent secondary antibodies. Fluorescent secondary antibody in NGB, DAPI, and PBS/T washes and mounting are as described above. Lamellipodia were quantified as cells exhibiting phalloidin staining as represented in Figure 6A, arrow. Peri-nuclear INTβ1 was quantified as cells exhibiting INTβ1 distribution as represented in Figure 6B, arrow.

### Live cell migration assay

Myoblasts were plated on laminin (Sigma) in a 12-well dish, 10,000 cells per well, and cultured overnight in 10% FBS, 1% Penn/Strep, DMEM (migration media). Prior to imaging, fresh migration media, with or without 4 ng/ml HGF [14] (Invitrogen) was added to wells. The β-GAL fluorescent substrate, Imagene Green (Invitrogen), was used according to the manufacturer’s suggested protocol. Images were captured every 4 minutes from 3 different fields in each well for 6 h, individual cell velocities were measured using Metamorph software. Migratory traces were based on Metamorph generated cell position data (x and y coordinates) and assembled using Microsoft Excel. 

### Fusion assay

For live imaging, myoblasts were plated on Matrigel in a 24-well dish, 13,000 cells/cm^2^. Prior to imaging, proliferation media was replaced with 2% FBS, 1% Penn/Strep, DMEM (differentiation media). Images were captured every 5 minutes for 48 h and processed using Metamorph.

For immunofluorescence, myoblasts were prepared from 2 mice each for control and mutant, and assayed independently (not mixed). Control and mutant cultures were grown in parallel for comparison. Cells were plated on Matrigel coated 8-well chamber slide at either 13,000 (low-density) or 49,000 (high-density) cells/cm^2^. Differentiation media was added to wells and cells were grown for 3 days, then fixed in 4% PFA, PBS. Differentiation index was determined by the number of nuclei in MHC+ YFP+ cells per nuclei in YFP+ cells. Index of unfused cells was determined by MHC+ YFP+ cells with single nucleus per total nuclei in MHC+ YFP+ cells. 

### Microscopy

Images of hematoxylin, eosin, and trichrome stained muscle sections were captured from a Nikon 800 microscope equipped with a 10x/.45 Plan Apo objective and Canon EOS T3 camera using EOS Utility image acquisition software. Fluorescent images (DAPI, Alexa Fluor 488, 568, and 647) of muscle sections were captured using either a Zeiss Axioscope equipped with a 20x/0.5 Plan Neofluar objective and Axiocam camera using Zeiss image acquisition software or Leica SP5 confocal equipped with 40x/1.25 and 63x/1.4 Plan Apo oil objectives using Leica image acquisition software. Images of cells on single fibers were captured using the Leica SP5 setup. Live cell imaging was done with a Nikon TE2000 10x ELWD using Hoffman Modulation Contrast (HMC) and a Photometrics Coolsnap HQ camera using Metamorph software. All images were processed using ImageJ64 software.

## Supporting Information

Figure S1 **c-MET is required for SC mediated muscle regeneration.** A) Control and mutant muscle tissue showing IF against embryonic MHC (green) and stained with DAPI (blue) in 5 dpi muscle sections (scale bar = 50 μm). B) Quantification of regenerated fiber number per 0.38 mm^2^ area in 10 dpi control and mutant muscle sections (p = .02 *t* test; N = 3 mice; 3 fields per mouse; error bars = SEM). C) Quantification of regenerated fiber diameter in 10 dpi control and mutant muscle sections (p = 5.76E-35 t test; N = 3 mice; 3 fields per mouse; error bars = SEM).
(TIF)

Figure S2 **Myogenic cells labeled with Imagene Green during live cell imaging.** HMC and epifluorescence (FITC filter) images of live cells at the beginning of movies (Video S1 – control cells, and Video S2 – mutant cells) used for migration velocity measurements. Control (*Pax7^CE/+^; c-Met^+/+^; Rosa26^LacZ^* ) and mutant (*Pax7^CE/+^; c-Met^F/F^; Rosa26^LacZ^*) cells are labeled with the β-GAL fluorescent substrate, Imagene Green (arrows). Non-myogenic cells do not label green (arrowhead; scale bar = 50 μm).
(TIF)

Figure S3 **Assessment of apoptosis in differentiating cultures.** A) IF for YFP and cleaved CASPASE-3 (CAS3) in 3 day differentiated cultures. (Arrows, YFP+ CAS3+ cells; scale bar = 100 μm). B) Average percentage of YFP+ CAS3+ per total YFP+ cells containing a single nucleus. (p = .033 *t* test; N = 3; n = 400 cells; error bars = SEM). C) IF for YFP, CAS3, and MHC in 3 day differentiated cultures. (Arrows, YFP+ CAS3+ MHC- cells; scale bar = 100 μm).
(TIF)

Video S1 **Migration of control myoblasts.** Images were analyzed by time-lapse, HMC microscopy using a Nikon TE2000. Frames were taken every 4 min for 6 hours and are played at a rate of 10 frames per second.
(MOV)

Video S2 **Migration of mutant myoblasts.** Images were analyzed by time-lapse, HMC microscopy using a Nikon TE2000. Frames were taken every 4 min for 6 hours and are played at a rate of 10 frames per second.
(MOV)
