# Supplementary figures and images for: c-MET Regulates Myoblast Motility and Myocyte Fusion during Adult Skeletal Muscle Regeneration
Source: PLoS One. 2013 Nov 19;8(11):e81757. doi: 10.1371/journal.pone.0081757 (PMC3834319; doi:10.1371/journal.pone.0081757)

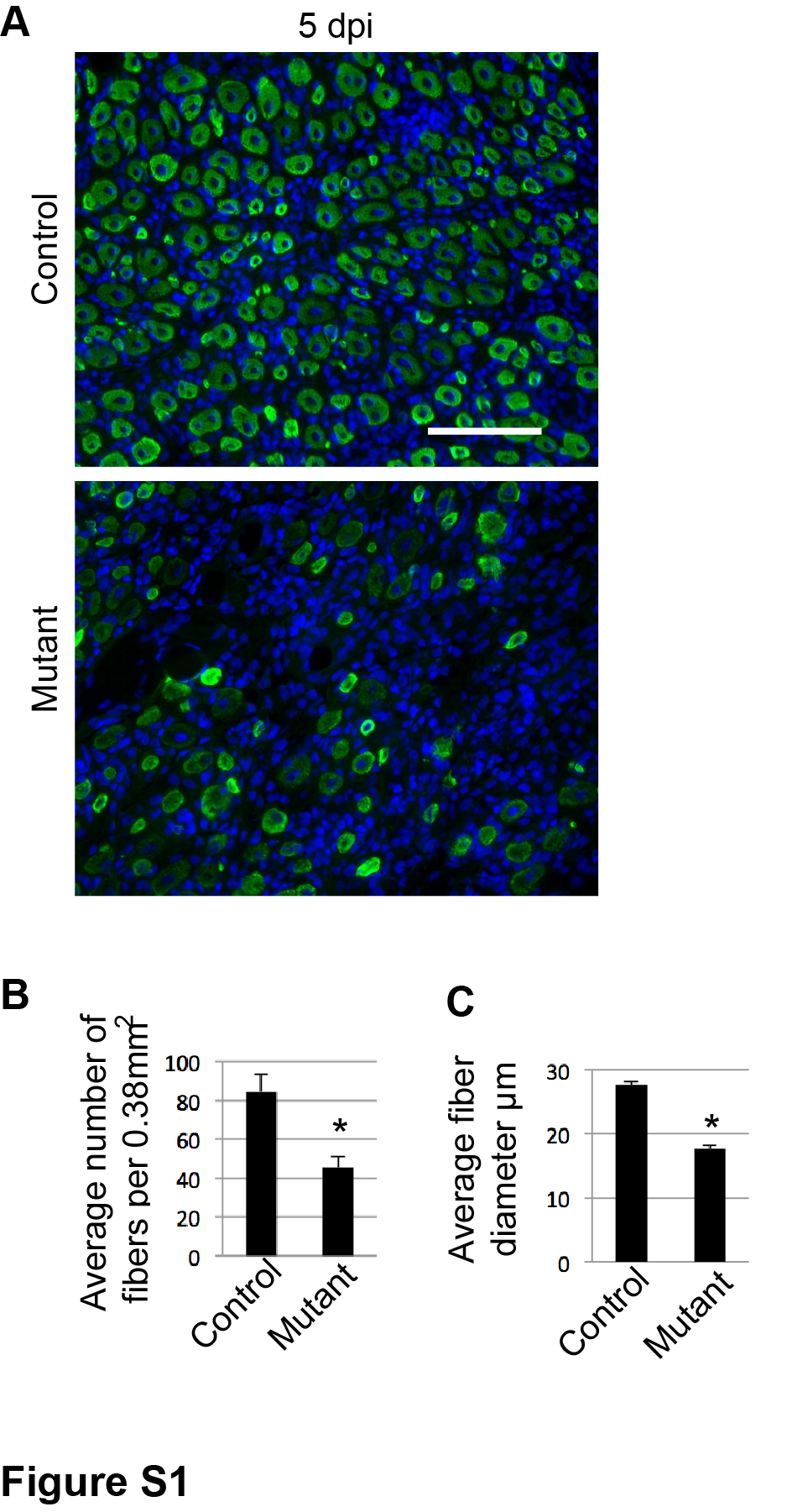

Supplement: Figure S1 — c-MET is required for SC mediated muscle regeneration. A) Control and mutant muscle tissue showing IF against embryonic MHC (green) and stained with DAPI (blue) in 5 dpi muscle sections (scale bar = 50 μm). B) Quantification of regenerated fiber number per 0.38 mm2 area in 10 dpi control and mutant muscle sections (p = .02 t test; N = 3 mice; 3 fields per mouse; error bars = SEM). C) Quantification of regenerated fiber diameter in 10 dpi control and mutant muscle sections (p = 5.76E-35 t test; N = 3 mice; 3 fields per mouse; error bars = SEM). (TIF) [file pone.0081757.s001.tif]

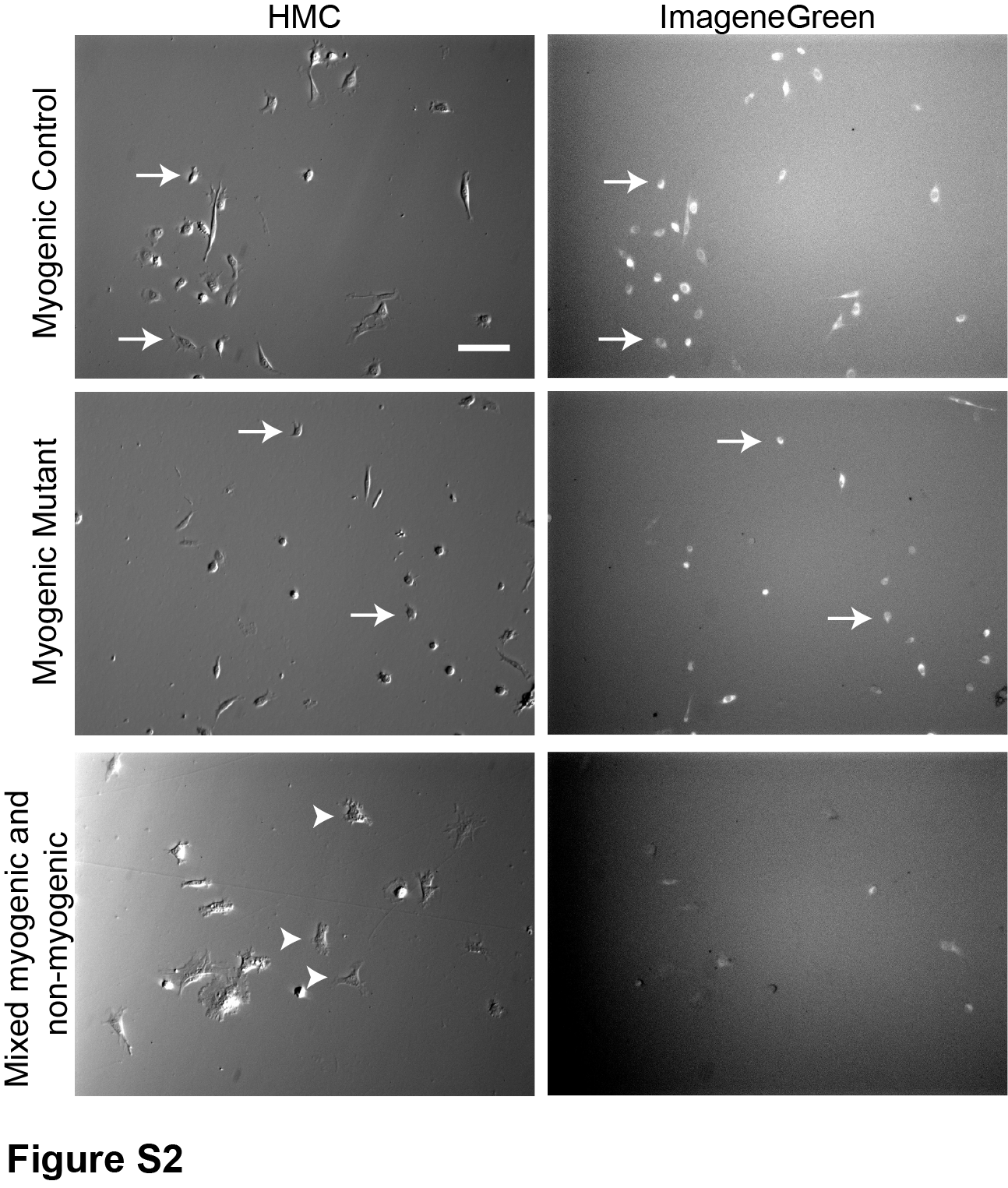

Supplement: Figure S2 — Myogenic cells labeled with Imagene Green during live cell imaging. HMC and epifluorescence (FITC filter) images of live cells at the beginning of movies (Video S1 – control cells, and Video S2 – mutant cells) used for migration velocity measurements. Control (Pax7CE/+; c-Met+/+; Rosa26LacZ ) and mutant (Pax7CE/+; c-MetF/F; Rosa26LacZ) cells are labeled with the β-GAL fluorescent substrate, Imagene Green (arrows). Non-myogenic cells do not label green (arrowhead; scale bar = 50 μm). (TIF) [file pone.0081757.s002.tif]

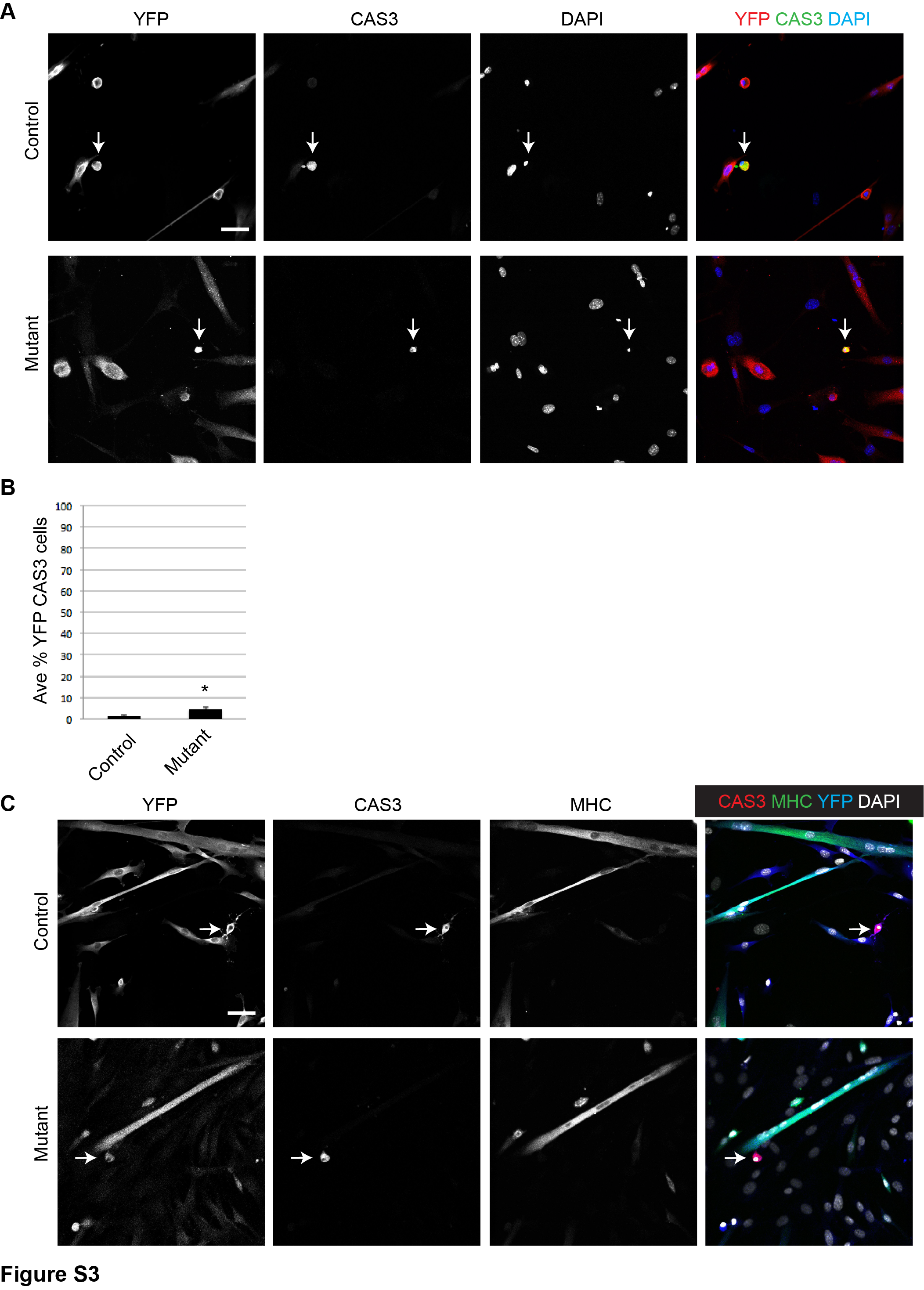

Supplement: Figure S3 — Assessment of apoptosis in differentiating cultures. A) IF for YFP and cleaved CASPASE-3 (CAS3) in 3 day differentiated cultures. (Arrows, YFP+ CAS3+ cells; scale bar = 100 μm). B) Average percentage of YFP+ CAS3+ per total YFP+ cells containing a single nucleus. (p = .033 t test; N = 3; n = 400 cells; error bars = SEM). C) IF for YFP, CAS3, and MHC in 3 day differentiated cultures. (Arrows, YFP+ CAS3+ MHC- cells; scale bar = 100 μm). (TIF) [file pone.0081757.s003.tif]
